# Supercapsular percutaneously assisted (SuperPath) approach in total hip arthroplasty: Surgical technique and preliminary results

**DOI:** 10.1007/s00064-019-0597-5

**Published:** 2019-04-12

**Authors:** H. Quitmann

**Affiliations:** Fabricius Klinik, Gelenkzentrum Bergisch Land, Freiheitstsr. 203, 42853 Remscheid, Germany

**Keywords:** Osteoarthritis, hip, Total hip replacement, Minimally invasive surgery, Cementless arthroplasty, Femoral neck fracture, Coxarthrose, Hüftprothese, Minimal-invasiv, Zementfreie Endoprothetik, Schenkelhalsfraktur

## Abstract

**Objective:**

Portal assisted minimally invasive total hip arthroplasty without dislocation of the femoral head with preservation of the hip capsule and the external rotators in the lateral decubitus position for rapid recovery with the option of expandability to a mini posterior or classic posterolateral approach at any time.

**Indications:**

Primary and secondary arthritis of the hip, femoral head necrosis, femoral neck fracture.

**Contraindications:**

Severe anatomical disorders of the proximal femur, congenital high hip dysplasia, implanted hardware in the trochanteric region, local and systemic infections.

**Surgical technique:**

Lateral decubitus position, skin incision of 6–10 cm from the tip of the greater trochanter in line with the femoral axis, spread gluteus maximus, using the interval between the piriformis tendon posterior and gluteus minimus/medius muscle anterior, incision of the capsule, remove bone of the lateral neck and head, intramedullary reaming and broaching of the femur, osteotomy of the femoral neck with the femoral broach left in situ, remove the femoral head, preparation of the acetabulum using a cannula posterior of the femur, cup impaction and implantation of the inlay, trial modular neck and head, reposition, test of leg length, impingement and stability, x‑ray, implantation of the definitive components, closure of the capsule, standard wound closure.

**Postoperative management:**

Full weight bearing as possible, no restrictions of postoperative movement.

**Results:**

The first 150 patients were operated from January 2016 to July 2017 without leg length discrepancy more than 5 mm; one transfusion was needed. There were two subluxations, one wound dehiscence and one femoral diaphyseal fracture 4 weeks after surgery. There was no radiological loosening of the components after a mean of 16 months.

## Zusammenfassung

**Operationsziel:**

Portalassistierte minimal-invasive Implantation von Hüftendoprothesen ohne Hüftkopfluxation mit Erhalt der Hüftgelenkkapsel und der Außenrotatoren in Seitenlage für eine schnelle Mobilisation mit der Option der Erweiterbarkeit in einen posterioren Mini- oder klassischen posterolateralen Zugang.

**Indikationen:**

Primäre und sekundäre Coxarthrose, Femurkopfnekrose, Schenkelhalsfraktur.

**Kontraindikationen:**

Erhebliche anatomische Fehlstellungen des proximalen Femur, kongenitale hohe Hüftluxation, einliegendes Osteosynthesematerial am proximalen Femur, lokale und systemische Infektionen.

**Operationstechnik:**

Seitenlage, Hautschnitt kranial des Trochanters von 6–10 cm Länge in Verlängerung der Femurachse, Inzision der Faszie des M. gluteus maximus und Spreizen der Muskelfasern, Eröffnen der Bursa am Hinterrand des M. gluteus medius, Nutzen des Intervalls zwischen der Piriformissehne dorsal und den Mm. gluteus medius und minimus ventral, Kapselinzision, Markraumeröffnung, Entfernung von Knochen am lateralen Kopf und Schenkelhals, Aufraspeln des Markraums bis zur geplanten Größe, Schenkelhalsresektion bei Belassung der Schaftraspel im Femur, Entnahme des Hüftkopfs, Präparation der Hüftpfanne durch eine Arbeitskanüle für die Bohrwelle dorsal des Femurs, Implantation der Hüftpfanne mit Inlay, Einsetzen von Probehals und -kopf, Probereposition, Überprüfung auf Beinlänge, Luxationsgefahr und Impingement, Durchleuchtung, Implantation des Originalschafts und -kopfs, Kapselverschluss, Wundverschluss nach Standard.

**Weiterbehandlung:**

Schmerzorientierte Vollbelastung ohne Bewegungseinschränkung.

**Ergebnisse:**

Die ersten 150 Patienten wurden zwischen Januar 2016 und Juli 2017 operiert. Eine Beinlängendifferenz von mehr als 5 mm trat nicht auf. Eine Transfusion war notwendig. Es traten zwei Subluxationen und eine Femurfraktur 4 Wochen postoperativ auf. Alle Komponenten sind fest eingewachsen bei einer durchschnittlichen Nachuntersuchungszeit von 16 Monaten.

## Introductory remarks

Total hip arthroplasty usually requires dislocation of the femoral head for osteotomy of the neck. This may lead to rupture of the external rotators in techniques with internally rotation of the femur as it is necessary with a classic or minimally invasive posterior approach [7]. With this minimally invasive technique it is possible to keep the femoral head and neck during the femoral canal broaching. The external rotators are preserved as well as the hip capsule. There are no unphysiological movements of the leg necessary. The technique was first described by Chow et al. [3]. This technique allows early mobilization with full weight bearing without restriction of movements.

## Surgical principle and objective

Implantation of a total hip arthroplasty with a muscle sparing minimally invasive approach without dislocation of the femoral head with preservation of the capsule.

Lateral decubitus position, skin incision of 6–10 cm from the tip of the greater trochanter in line with the femoral axis, incision of the fascia of the gluteus maximus muscle, blunt dissection of the fibers, incision of the bursa at the posterior boarder of the gluteus maximus muscle, using the space between the piriformis posterior and the gluteus minimus and medius muscle anterior, incision of the capsule, opening of the femoral canal with a starter reamer, creating a channel in the corticalis of the lateral neck up to the lateral part of the head with a round calcar punch, sequentially broaching of the femur, osteotomy the femoral neck at the tip of the femoral broach left in situ, removal of the femoral head, preparation of the acetabulum, use of a cannula posterior of the femur to pass the reamer drive shaft, connecting the acetabular basket reamer through the main incision, cup impaction and implantation of the inlay, trial modular neck and head, reposition, intra-operative radiograph, test of leg length, impingement and stability, implantation of the definitive components, closure of the capsule, standard wound closure.

## Advantages

- Short incision of 6–10 cm
- Preservation of the external rotators
- Preservation of the capsule
- No use of curved instruments necessary
- No special table required
- Avoiding non physiologic leg manipulations

## Disadvantages

- Limited view to the proximal femur

## Indications

- Primary and secondary osteoarthritis of the hip
- Femoral head necrosis
- Femoral neck fracture

## Contraindications

- Relative:
  - Severe deformation of the proximal femur
  - Congenital high hip dysplasia
  - Osteosynthesis of the proximal femur
- Absolute:
  - Local or systemic infection
  - Open wounds in area of incision
  - Planned femoral osteotomy

## Patients information

- General surgery related risk factors (infection, bleeding, nerve injury)
- Risk of a transfusion (very low)
- Fracture of the trochanter of the femur
- Dislocation of the hip
- Leg length discrepancy
- Implant loosening requiring revision surgery
- Implant wear requiring revision surgery
- Estimated stay in hospital 2–7 days
- Full weight bearing depending on pain allowed
- Return to work and activities depending on work

## Preoperative work up

**Fig. 1:**
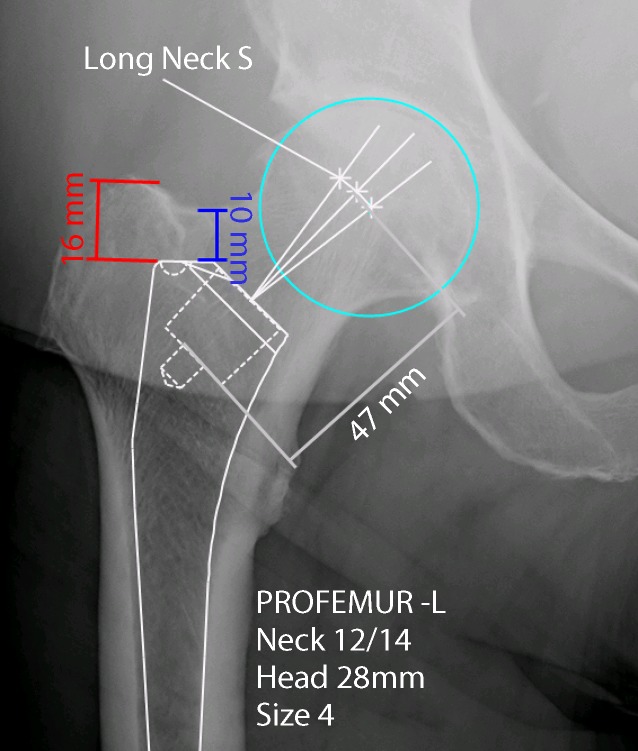
Digital planning preoperative. Cranial anterior neck distance (*blue*), trochanter distance (*red*), distance between the depth of the broach pocket and the middle of the femoral head (*grey*), the *turquoise circle* determines the center of the femoral head

- Clinical assessment with exclusion of infection
- Radiographic evaluation and digital planning of the components:
  - measurement of the distance between the cranial anterior neck (blue) or of the trochanter (red) and the shoulder of the prosthesis (Fig. 1)
  - Measurement of the distance between the depth of the broach pocket and the middle of the femoral head (grey) (Fig. 1)
  - Offset und reconstruction of the leg length
- Decolonization of the skin before surgery according to the standard protocol of the institution
- Local shave just before the surgery
- Routine antibiotic prophylaxis 30 min prior to the skin cut
- Tranexamic acid according to the standard protocol of the institution

## Instruments and implants

- Basic surgical instrument set
- Instruments for preparation of the femur and the acetabulum and implants required from Microport Orthopedics (MicroPort Orthopedics, Inc., Arlington, TN, USA). Uncemented and cemented implants can be used.
- Oscillating saw
- Cork screw

## Anesthesia and positioning

- General anesthesia
- Routine operating table
- Lateral decubitus position
- No traction device
- Intraoperative fluoroscopy not necessary but reasonable

## Surgical technique

(Figs. 2, 3, 4, 5, 6, 7, 8, 9, 10, 11, 12, 13, 14, 15).

**Fig. 2:**
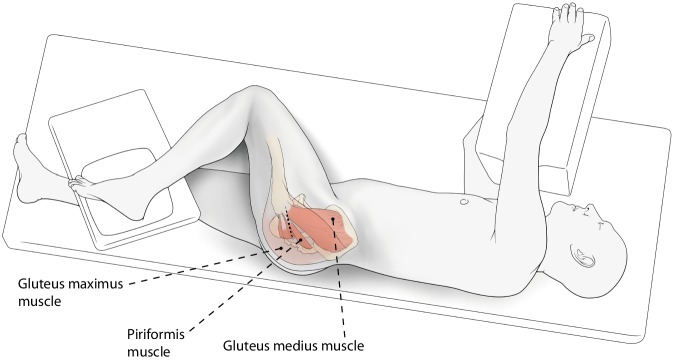
*Patient positioning. *Patient is in the lateral decubitus position with the operated leg in approximately 60° of flexion, and 20 to 30° of internal rotation in maximal adduction. The foot lays on a small pad or a mayo stand (the “home” position). Standard disinfection and draping with the operated leg free to move

**Fig. 3:**
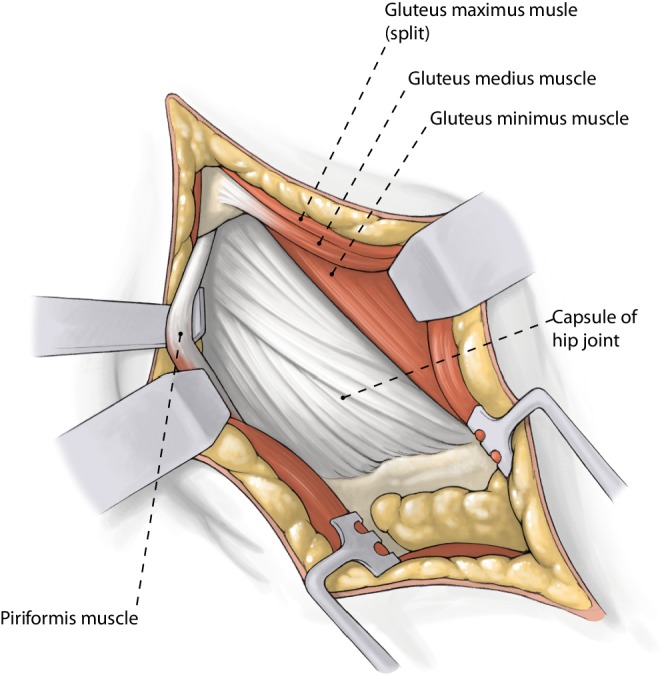
*Approach to the capsule. *Skin incision from the tip of the trochanter 6–10 cm proximally in line with the femur. Incise subcutaneous fat with electrocautery to reduce bleeding and fascia of the gluteus maximus muscle in line with the skin incision. Split the fibers of gluteus maximus with the wing tipped elevators—coagulation of crossing vessels to avoid bleeding. Incise bursa tissue at the posterior rim of gluteus medius. Retract the gluteus medius anterior and identify the piriformis tendon. Use the Cobb elevator to dissect and move the gluteus minimus anterior. Place sharp retractor under the piriformis tendon posterior (at this time elevate the knee) and another one under the gluteus medius anterior. You can now clearly see the capsule. Place a Zelpi retractor in the proximal part of the capsule. A release of the piriformis tendon is possible at any time to alleviate the exposure to the capsule and acetabulum or to treat external contracture

**Fig. 4:**
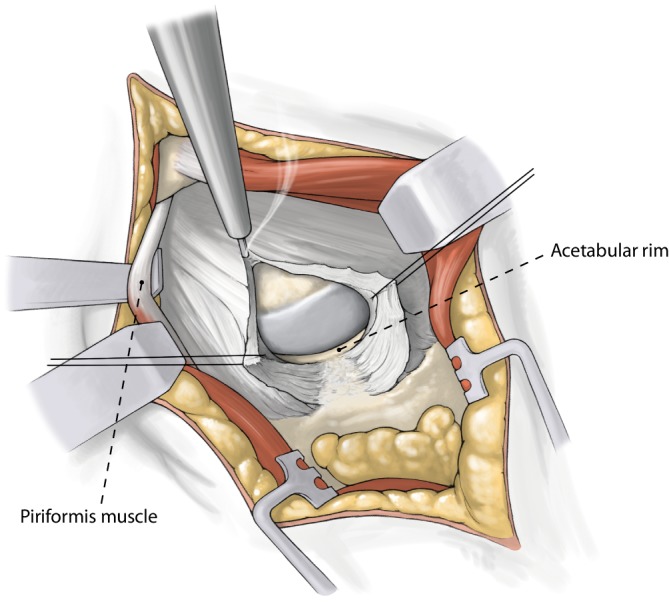
*Intracapsular preparation. *Capsular incision is made in line with the skin and the femoral neck with an electrocautery. Free the lateral border of the acetabulum 1 cm anterior und posterior. Remove a part of the labrum. Hemostasis is important at the basis of the capsule. Replace the sharp elevators with blunt Hohmann around the neck first posterior then anterior. Identify the saddle of femoral neck (*green line* in Fig. 7)

### Femoral preparation

**Fig. 5:**
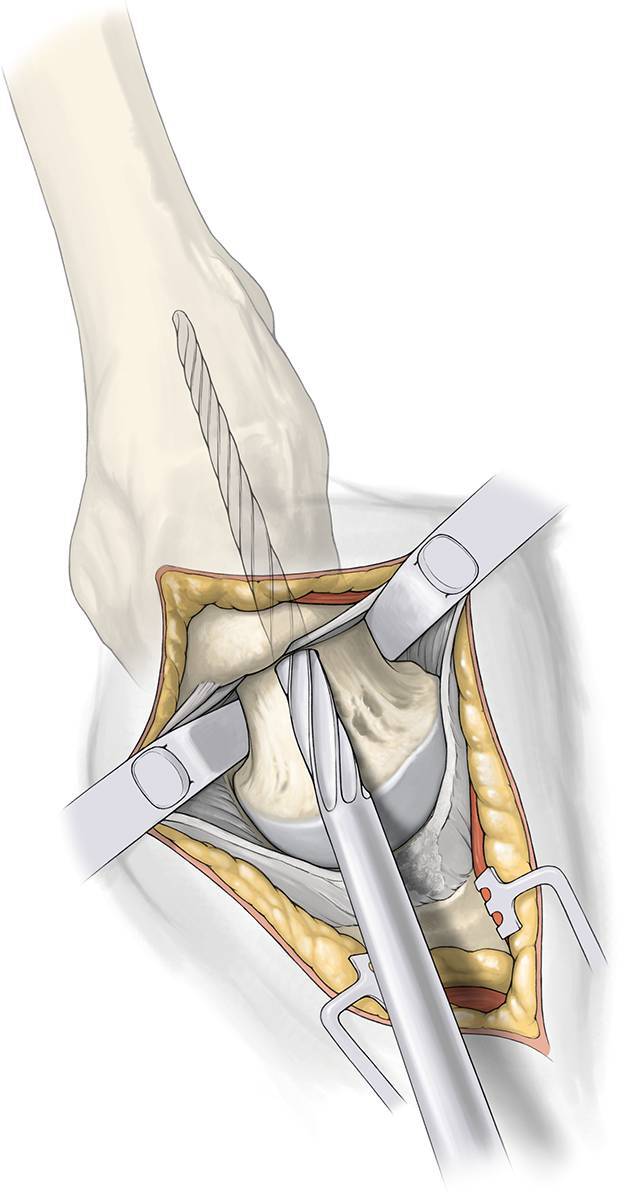
Start femoral preparation with head and neck intact. Opening of the femoral canal with a sharp starter reamer. Confirm intramedullary reaming with a cortical feeler gauge beyond the level reamed. Use a blunt metaphyseal reamer with pressure against the medial part of the trochanter to get in line with the femoral canal

**Fig. 6:**
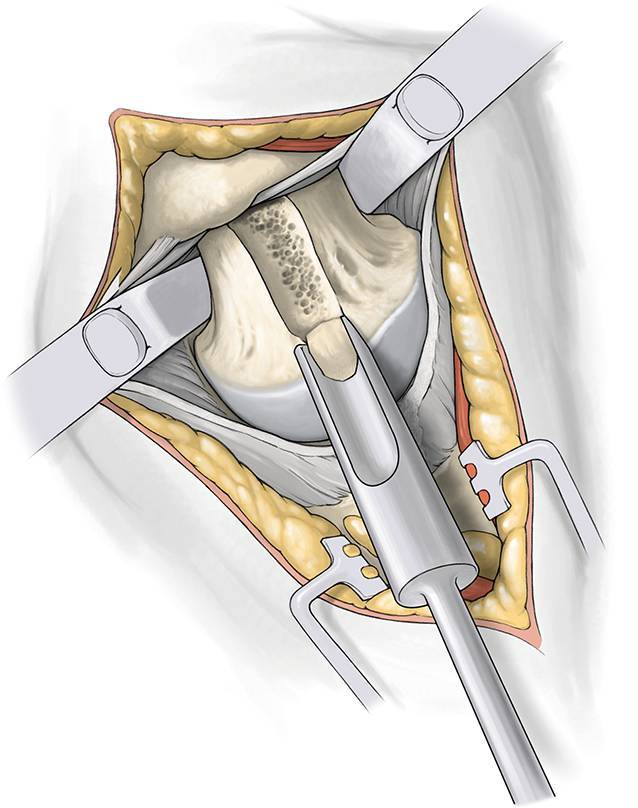
Remove a small part of bone from the lateral part of the femoral head for passing the femoral broach using a round calcar punch. This is important for the anteversion of the femoral component. The femoral metaphysis is reamed with the broaches until metaphyseal contact always comparing with the digital planning

**Fig. 7:**
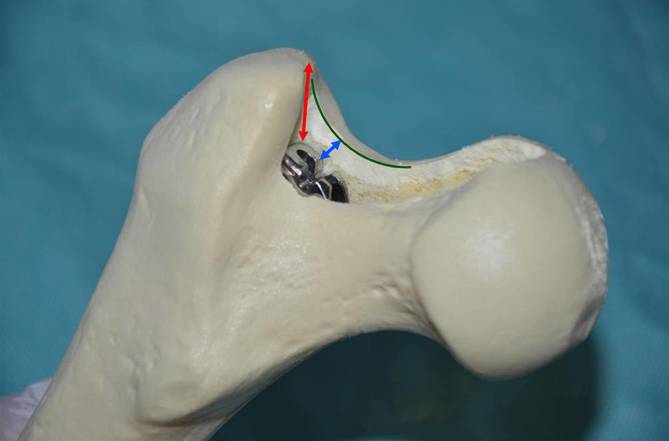
The correct depth of the broach can be measured. You can clearly see the anterior part of the neck (*green line*) and feel the distance to the proximal end of the broach (*blue arrow*). Alternatively measure the difference between the tip of trochanter to the shoulder of the broach (*red arrow*). Leave the broach inside. Cranial anterior neck distance (*blue*), trochanter distance (*red*), anterior part of the neck (*green*)

**Fig. 8:**
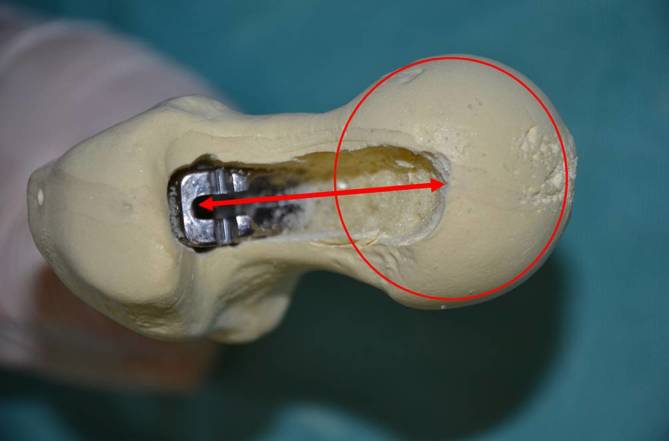
To check femoral offset one can measure the distance between the middle of the femoral head to the broach pocket

**Fig. 9:**
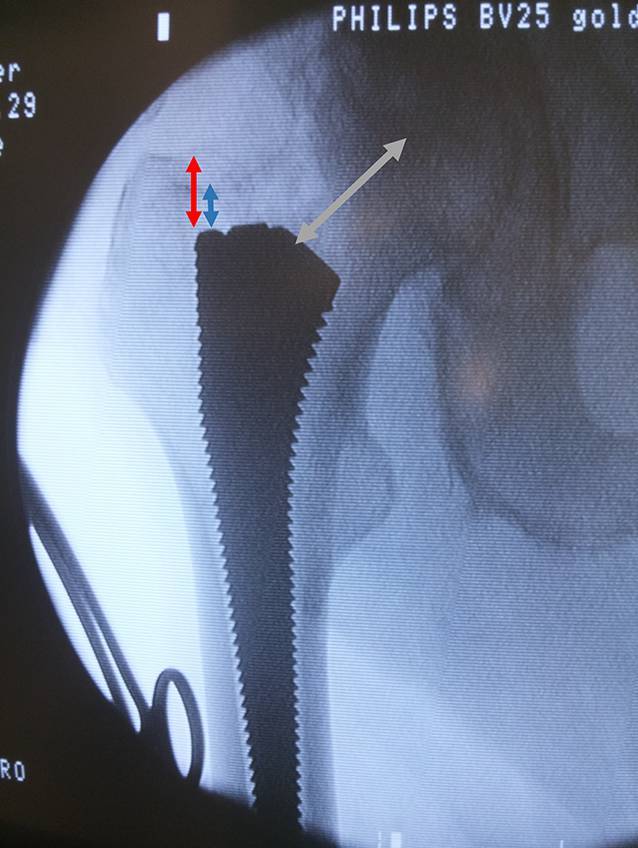
If desired compare intraoperative radiographic evaluation with the c‑arm and digital planning of the components

(Figs. 5, 6, 7, 8, 9).

**Fig. 10:**
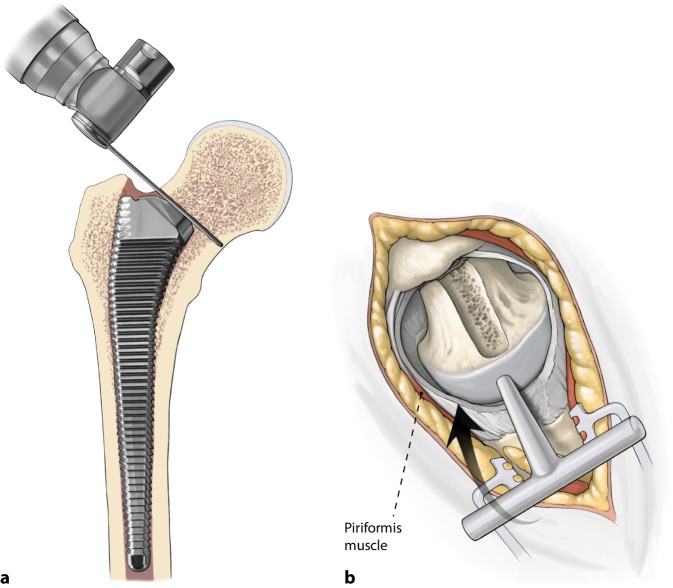
**a** and** b** *Femoral head resection and removal.* Osteotomy of the femoral neck is performed with the broach as a saw guide (**a**). The foot is lifted to check the complete osteotomy. A cork screw is used for removal of the femoral head (**b**). Take away blunt Hohmann retractors. The leg is distended by the assistant in 30° of flexion, slight abduction and neutral rotation to release the tension of the capsule. The head is then adducted as much as possible to rupture the ligamentum teres. The head is pulled out. Have a look at the anterior border of the femur with a slight internal rotation of the femur. Osteophytes are removed to prevent impingement with the anterior acetabulum in flexion. The leg is brought back to the home position

**Fig. 11:**
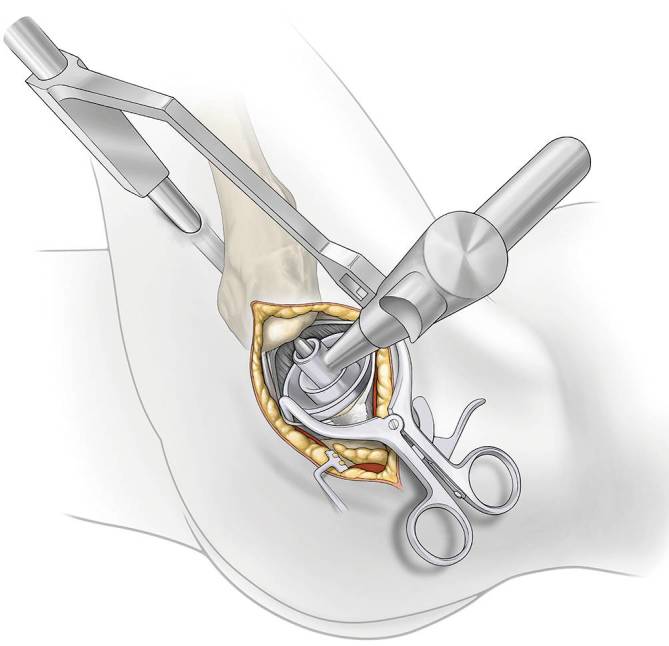
*Acetabular exposure. *For exposure of the acetabulum a Romanelli retractor is placed inside the capsule just at the anterior and posterior wall or sharp Hohmann retractors are applied between the labrum and the bony edge. The rest of the labrum is removed and the transverse ligament is identified. There should be no bleeding from the ligamentum capitis femoris. A bone hook is placed into the shoulder of the femoral broach (avoid to place the hook into the trochanter) to retract the femur anterior if necessary. An alignment tower is seated in the acetabulum. The leg is placed in extension with a flexed knee to release tension of the sciatic nerve and palpate the femur. Then, 1–2 cm posterior to femur set a 1 cm incision to allow a cannula to pass inside the capsule just posterior the femoral neck. The alignment tower is removed leaving the cannula in place. The leg is brought in slight flexion and external rotation to relax the capsule for a better exposure of the acetabulum. The anterior and posterior wall should be seen as well as the transverse ligament and the fossa acetabula for optimal positioning of the cup

**Fig. 12:**
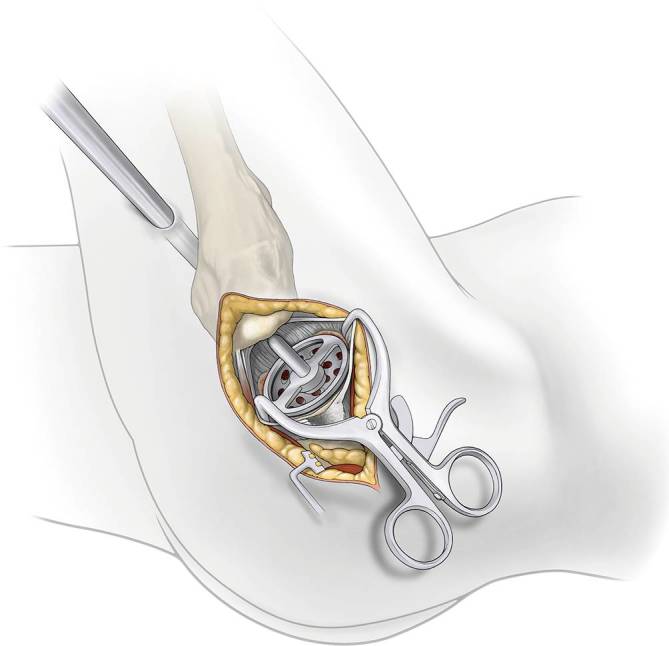
*Acetabular preparation. *The reamer shaft is passed through the cannula and is mated with the acetabular reamers inside the capsule. A reamer basket holder is used to pass the reamer through the main incision. The leg can be moved to assist in proper angle for optimal anteversion. Direct visualization of the entire acetabulum through main incision is always possible

**Fig. 13:**
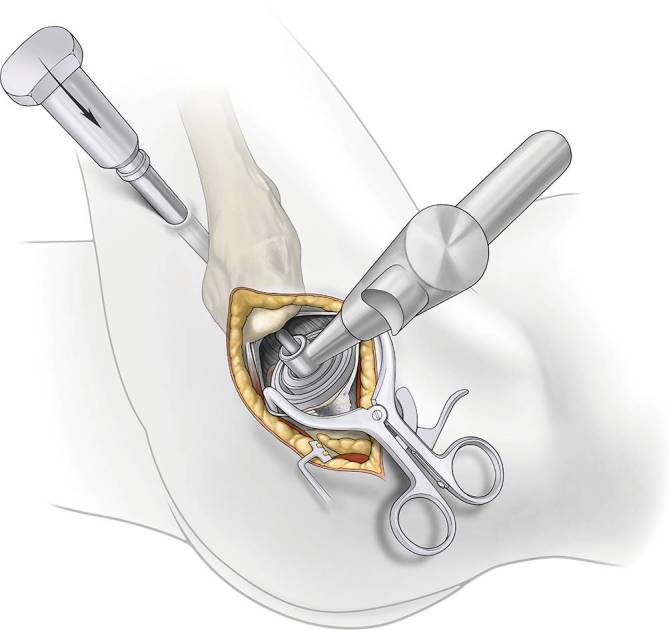
*Acetabular cup placement. *Attach threaded cup adapter to cup and alignment handle. Impact to medialize cup. Insert cup impactor to engage cup in 40° of abduction and natural anteversion parallel to the transverse ligament regarding the anterior and posterior wall. Fixation of the cup with screws is now possible through the cannula if needed. A trial liner is inserted or the final liner using the liner impactor and the osteophytes of the acetabulum are removed as well as the cannula

**Fig. 14:**
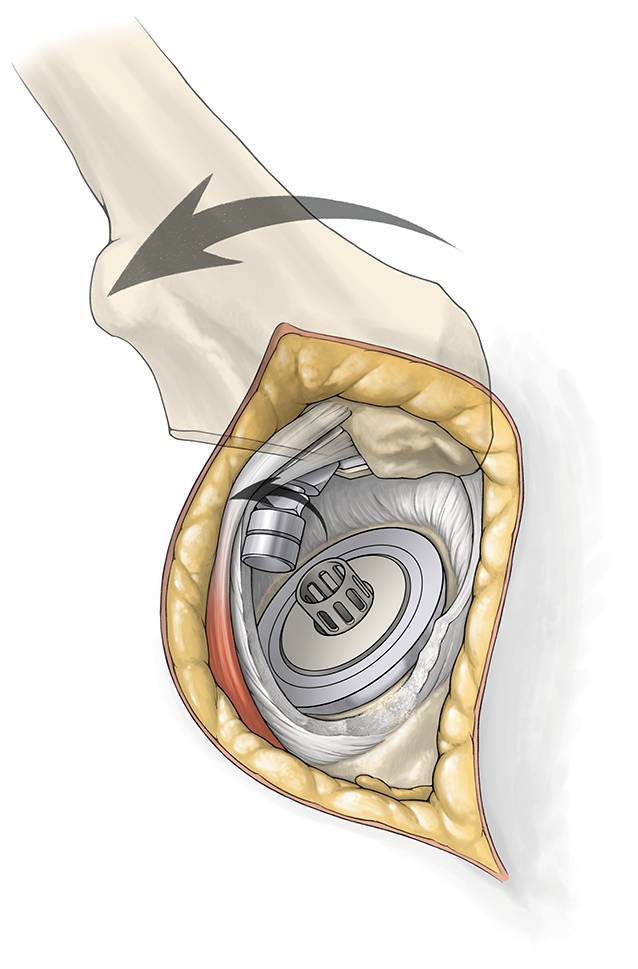
*Trial reduction. *A trial head is inserted into the liner and a trial neck according to the preoperative planning into broach pocket. A blunt trocar is applied against the top of broach to mate. The assistant moves knee or foot while surgeon moves the leg to complete the reposition. If the blunt trocar is used in one hand and the reamer basket holder in the other hand, guide the trial neck to “mate” the trial head inside of the acetabulum. Leg length, stability (anterior, posterior, and lateral), range of motion, and impingement are checked. If there is still a flexion contracture release the capsule from the anterior femur and/or the anterior acetabulum. X‑ray can be performed to match result with the planning. X‑ray after trial reposition is not necessary but reasonable. Check the position of the acetabular component as well as the size und position of the femoral broach. Intraoperative fluoroscopy is easier using a padded peg board and radiolucent positioning pegs

**Fig. 15:**
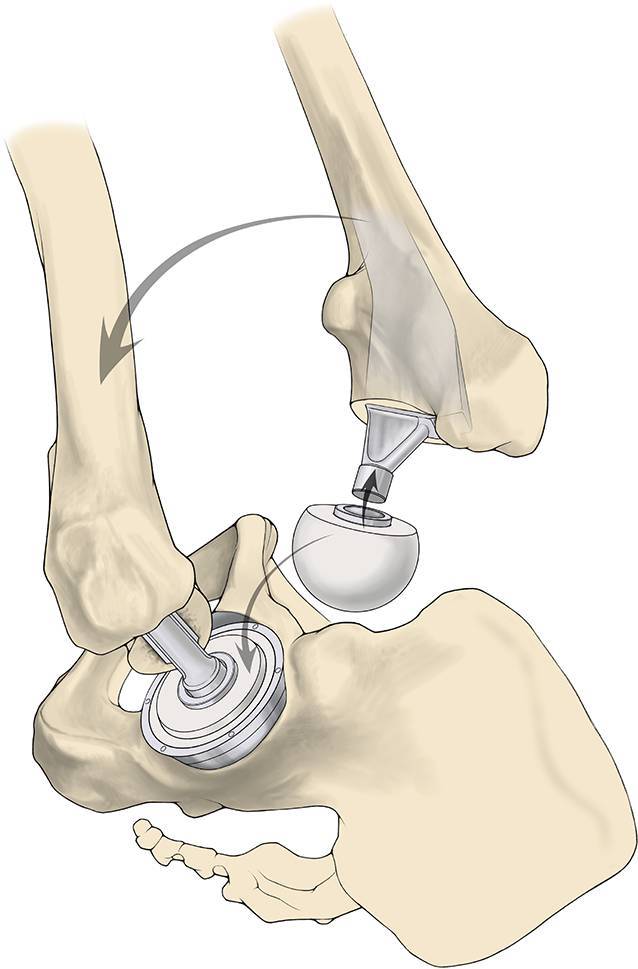
*Implant assembly. *To remove neck/head trials, leverage bone hook and blunt trocar are used or “classic” luxation to posterior–superior. Associated tapers are dried and cleaned. A modular or a monolithic stem is impacted into the femur. The head is placed on the neck in a classic manner or neck and head are connected inside as it is described above with the trial neck and head. The assistant moves knee or foot while the surgeon moves the leg to complete. Stability is checked again

### Wound closure

The wound is irrigated and checked for bleedings. Closure of the capsule with a running suture or single stitches. Reattachment of the piriformis tendon at the posterior border of the gluteus medius if release was necessary. No drain necessary but possible. Closure of the gluteus maximus fascia. Routine closure of subcutaneous tissue and skin. Spica for compression of the wound possible. Final x‑ray in a supine position is recommended before the patient leaves the operating room.

## Special surgical considerations

In our experience, this approach was beneficial for obese patients. There is not necessarily a need for a very long incision because the procedure is done in a “slot” with a minimal dissection of subcutaneous tissue.

Treating patients with a very stiff hip, it may be difficult to get the internal rotation as it is described above. In those cases, tilting the pelvis to get more adduction for the approach is helpful. There should be a space between the lateral boarder of the acetabulum and the medial part of the trochanter to pass the femoral broaches. This can be difficult for patients with a protrusion or a short (varus) neck. After preparation of the femur, return to a straight position for the acetabulum, essential for a correct positioning for the cup. Another possibility is to do a release of the capsule on the femoral side as well as on the acetabulum. Nevertheless, in most of the cases the closure of the capsule is possible.

Removal of all osteophytes at the anterior boarder of the neck is essential to prevent impingement especially in patients with a short neck.

Monolithic, modular, or cemented implants may be used according to the planning and personal or preferences.

In case of femoral neck fracture, it is also possible to use this approach leaving the femoral head in situ until preparation of the femur is finished. There is one publication about using the SuperPath approach for hemiarthroplasty [1].

With increasing experience, this approach can also be used for revision cases. There is the option to release the dorsal capsule or the external rotators at any time to extend the field of vision of the femur as required.

## Postoperative management

- Wound dressing is changed if necessary
- Full weight bearing allowed as tolerated by pain
- Active and passive motion without restriction to treat muscle contractures and restoration of range of motion with physiotherapy
- Venous thromboembolic prophylaxis for 5 weeks after surgery according to the national guidelines
- Radiographic assessment after surgery according to the national guidelines

## Errors, hazards, complications

- Malpositioning of the components: intraoperative correction after x‑ray
- Damage of the sciatic nerve: immediate release of any wound dressing, computed tomography for exclusion of hematoma and revision if needed.
- Fracture of femur: cerclage wiring, exchange of the femoral component
- Fracture of trochanter: tension band wiring and partial weight bearing for 6 weeks
- Early joint infection: debridement and lavage, exchange of liner and head, antibiotics

## Results

**Fig. 16:**
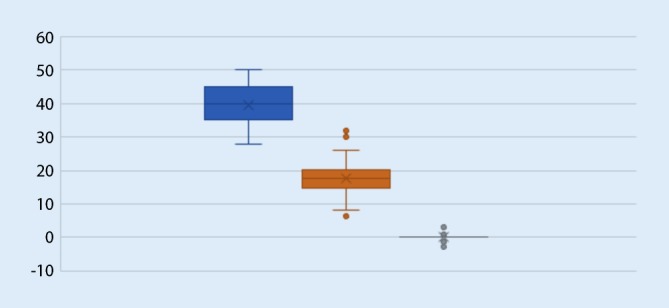
Box plots. *Blue*: cup inclination, *orange*: cup anteversion, *grey*: stem alignment

From January 2016 to July 2017, a total of 150 patients were treated with cementless total hip arthroplasty because of osteoarthritis of the hip. The average age at surgery was 69 years (range 39–86 years, 98 women and 52 men) and the average body mass index was 27 (range 17–48). The operative time was overall 81 min (range 58–121 min) declining from the first 50 patients (89 min) to the last 50 patients (75 min). There was a prospective follow-up. The inclination angle was at 39.3 (range 28–50) and there was no leg length difference more than 5 mm. The mean anteversion angle measured at a standard supine anteroposterior pelvis view was at 17.1 (range 6.2–31.9, SD 4). The position of the stem was 0.17° varus (range 2.7 valgus to 3.3 varus, SD 0.9) measured between the stem axis and the long axis of the femur (Fig. 16).

Postoperatively all the patients were mobilized early and discharged at day 9.9 (due to regulations of the hospital). Most of the patients were able to leave the hospital earlier.

Surgical complications occurred in 4 patients but only the first was related to the surgical technique. There were two subluxations: in case number 4 three weeks after surgery during elevated sitting. This woman with a body mass index of 34 underwent a closed reduction and is doing fine one year after surgery. The second subluxation in patient number 6 occurred in the operating room after turning the patient onto the back. This required immediate exchange of the head to a longer one. This demented patient walked without crutches after a few days without any complaints. In both cases there was no complete dislocation because the head was captured by the closed capsule.

We encountered one wound dehiscence in case 32 in a woman with a body mass index of 48 which required a new skin closure in the operating room at day 8. This was related to a new skin suture technique and not to the approach.

One patient who was 78 years old (case 132) was retransferred from the rehabilitation clinic after 4 weeks because a femoral diaphyseal fracture required cerclage wiring of the femur and exchange of the stem.

The radiologic data are similar to a short published study with a cup abduction angle of 43.6 ± 6.5, a cup anteversion angle of 17.4 ± 1.8 and a neutral stem alignment with no outliers of more than 5° [9]. The operative time was longer with 103.6 ± 11.8 min and the length of stay (8.3 days) was comparable. We measured the operative time including all patients starting from the beginning.

Rasuli and Gofton compared their first 50 patients with the PATH technique [5, 6]. They had an operative time in the SuperPath group of 101.7 min (SD 18.3), a complication rate of 4%, an acetabular abduction angle of 39.0° ± 8.4°, and an anteversion angle of 23.5° ± 8.2°. The anteversion angle was higher in the SuperPath group than in the PATH group requiring more use of the transverse acetabular ligament as a guide to reduce this effect [6].

In another study postoperative radiographs of 66 consecutive patients from the first 100 patients were measured. They found a mean acetabular abduction angle of 40.13° ± 6.30° [4].

In a smaller series of the first 21 patients, the average operating time was 102.85 min (range 80–130 min). The mean acetabular inclination was 44.05° (26–60°) [2].

The results of another study showed similar results with a low complication rate and excellent patient satisfaction [8].

## Abbreviations

SD: Standard deviation
